# Gas Turbine Blade Characterization Through Modal Analysis

**DOI:** 10.3390/ma19061192

**Published:** 2026-03-18

**Authors:** Andrea Troglia Gamba, Francesco Bagnera, Daniele Botto

**Affiliations:** 1 Department of Mechanical and Aerospace Engineering, Politecnico di Torino, 10129 Turin, Italy; s329645@studenti.polito.it; 2 Ansaldo Energia, 16152 Genoa, Italy; francesco.bagnera@ansaldoenergia.com

**Keywords:** gas turbine blade, rib turbolators, material optimization, conventionally solidified materials, directionally solidified materials, vibrations, frequency assessment, young’s module, deviations

## Abstract

This study presents the dynamic characterization of a gas turbine blade manufactured from two different nickel-based superalloys: on the first hand, a superalloy called René 80 and, on the second hand, a directionally solidified (DS) nickel-based anisotropic superalloy, investigated during the validation phase of the development process. Starting from the original CAD geometry, precise and very detailed finite-element models were developed, progressively refined and modified, and consequently validated to ensure mesh-independent modal predictions. The study examines multiple possible sources of discrepancy between experimentally measured and numerically predicted natural frequencies, including geometric deviations, grouping of different interesting points, broach-block test configuration, material anisotropy, and the influence of internal rib turbulators. Statistical analyses of dimensional variations revealed no significant correlation with the observed frequency scatter, redirecting the investigation toward material behavior and modeling fidelity. The inclusion of turbulators in the finite-element model proved essential, reducing prediction errors for the first two modes by approximately 2–3%. For the DS superalloy, the effect of grain orientation was evaluated over permissible angular deviations (extremes were considered); however, no systematic and clear improvement in frequency prediction was observed. Finally, several tuning strategies were assessed, leading to an optimization procedure that simultaneously adjusted the elastic moduli $E_{x}$ and $E_{z}$, reducing modal frequency deviations to below 1% for the first two modes. The proposed methodology provides a robust and solid framework for the validation of turbine blade dynamic behavior across different materials and manufacturing conditions.

## 1. Introduction

Modern gas turbine blades are critical components of complex turbomachinery systems and therefore require rigorous testing and validation before entering service. Operating under extreme thermal and mechanical conditions, turbine blades are subjected to significant dynamic loads, making accurate assessment of their vibrational behavior fundamental for ensuring structural integrity and operational reliability. In particular, modern gas turbine rotors often operate at or near supercritical speeds, where even small unbalances may induce severe vibration levels, potentially leading to damage or failure if not properly controlled [1]. Other sources of vibration, such as external excitations, elastic deformations of supporting structures, and time-varying bearing forces, may further contribute to the overall dynamic response. Furthermore, vibrations can cause fretting [2], resulting in wear [3] or fatigue [4] damage. These effects are not considered within the scope of the present study. Dynamic stresses associated with vibrational phenomena are widely regarded in both industrial practice and the scientific literature as one of the primary contributors to catastrophic failures in gas turbine components [5]. Consequently, even minor inaccuracies in the prediction of vibrational behavior during the early design and validation phases may propagate into significant structural issues during operation. Since mechanical vibrations cannot be eliminated during service and may induce substantial cyclic stresses, their effects must be thoroughly investigated and reliably predicted [6]. In this context, accurate determination of blade natural frequencies and mode shapes represents a fundamental requirement in turbine blade development. Similar conclusions have also been reported in high-performance blade applications beyond gas turbines, where finite-element studies on advanced material-reinforced wind turbine blades have demonstrated that material composition and structural modeling significantly influence static bending, free vibration, and torsional behavior when validating numerical predictions against experimental observations [7]. These findings further underline the critical role of material representation and modeling fidelity in blade dynamic analysis. In current industrial practice, experimentally measured natural frequencies are routinely compared with predictions obtained from finite element (FEM) models implemented in commercially available software, in order to assess the fidelity of numerical simulations against real-world behavior [8]. Modal analysis, followed by experimental validation, therefore constitutes a well-established step in gas turbine blade design, as vibrational integrity represents a critical design constraint and excessive forced-response amplification may result in catastrophic failure [9]. Numerical modal analysis is extensively employed to support experimental campaigns, reducing the number of required test iterations while providing a reliable starting point for experimental investigation [10]. Experimental characterization of structural dynamic behaviour has consistently proven essential for understanding vibration mechanisms and predicting in-service performance. As a result, experimental modal analysis remains the primary reference for validating numerical predictions of blade dynamics [11]. Even beyond the initial design phase, reliable turbine blade diagnostics require accurate identification of operational frequencies and associated mode shapes, which in turn depend on robust correlation between experimentally derived vibration data and FEM-based modal predictions [12]. Several experimental techniques for modal testing are reported in the literature, including impact testing combined with acoustic measurements [13], excitation using vibration shakers [14], and oblique hammering methods [15]. Despite this variety of available approaches, impact hammer testing remains the industry standard for gas turbine blade applications due to its simplicity, repeatability, and effectiveness and is therefore adopted in the present work. Beyond experimental technique selection, recent literature also highlights the increasing material complexity of turbine blades and the need for comprehensive characterization of both manufacturing variants and post-service materials. For instance, studies on the recycling and repurposing of wind turbine blade composites have shown that processing history and material treatment can significantly alter structural properties, with potential implications for both static and dynamic responses in service or laboratory validation contexts [16,17]. The comparison between experimentally measured and numerically predicted natural frequencies provides a direct means of validating FEM models for turbine blades and highlights their sensitivity to boundary conditions and test configurations [18]. In the specific case of hollow blades, internal geometric features play a particularly significant role in the dynamic response. As a result, careful identification of discrepancies and their underlying causes is required, as hollow blades may exhibit dynamic behaviour that differs substantially from that of solid configurations [19]. Despite continuous advances in modeling techniques, discrepancies between experimentally measured and FEM-predicted natural frequencies are still widely reported in the literature [20]. Lower-order modes are generally captured with higher accuracy due to their greater vibrational energy content, whereas higher-order modes often exhibit larger deviations [20]. Recent experimental–numerical studies on gas turbine blades continue to report residual discrepancies on the order of 3–5%, even when high-fidelity finite element models are employed [10]. Within this context, the methodologies discussed in the present work are investigated to systematically identify, quantify, and reduce potential sources of error in blade dynamic characterization.

## 2. Methodology

### 2.1. Work Flow Chart and Mesh Independence

**Figure 1:**
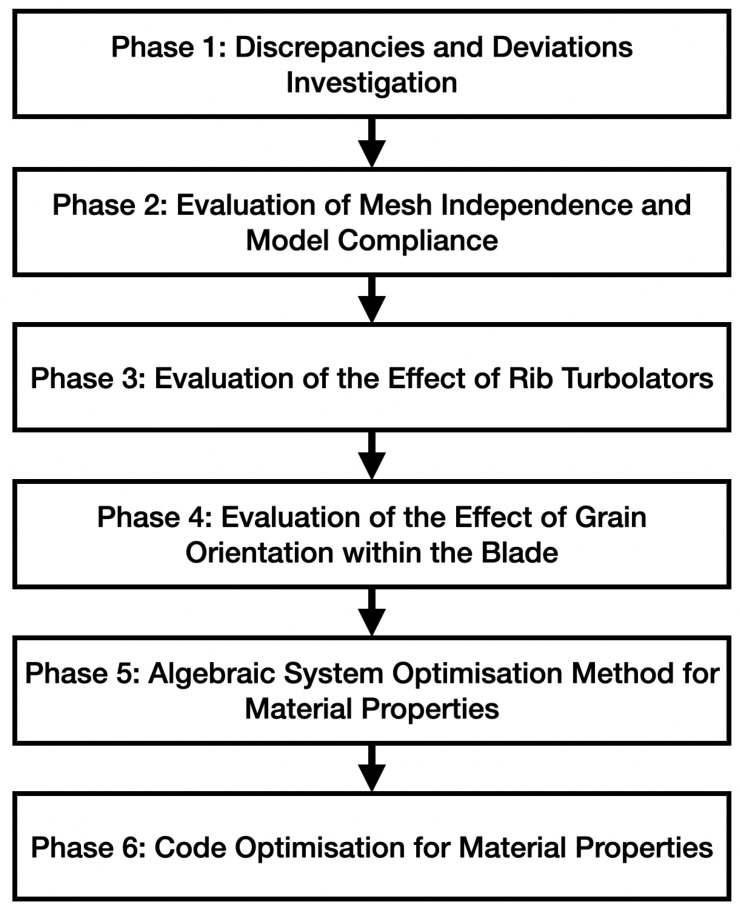
A Flow Chart to show at a glance the framework and overview of the presented work.

Before analyzing the values of the calculated frequencies, it was necessary to assess the complete fidelity of the mesh and the subsequent independence of results from the meshing process. In order to assess it, a series of meshes was developed, with very different numbers of points and elements. Even if a mesh independence graph were showing that after eight hundred thousand points, mesh-independent results were already being obtained, it was decided to continue calculations with one point six million elements, in order to be as close to reality as possible with the model. In addition, Figure 1 shows a simplified flowchart for an easier understanding of the presented following work. Each phase highlighted in the flowchart consisted of the initial idea and a follow-up in order to assess how results were obtained.

### 2.2. Statistical Characterization of Geometrical Deviations

The geometrical variability introduced during the manufacturing process of turbine blades can influence their structural dynamics and, consequently, their modal response. To quantify this variability and assess its potential correlation with measured natural frequencies, a statistical analysis was conducted on a batch of blades produced in both René 80 and directionally solidified (DS) nickel-based superalloys. Dimensional deviations were evaluated at predefined reference points located on the pressure (concave) and suction (convex) sides of each blade, following the metrology procedure adopted in production. The deviation from the nominal values refers to the thickness of the profile when compared to the nominal design value. For each measurement point, the deviation from the nominal CAD value was computed and aggregated over all inspected blades. This enabled the construction of empirical deviation distributions, which were subsequently compared to a theoretical normal distribution. The objective of this analysis was twofold: (1) verify that all blades complied with the dimensional limits specified by Ansaldo Energia, and (2) identify whether specific geometric deviations—or grouped deviations in selected blade regions—could explain the dispersion observed in experimental modal frequencies. All inspected blades fell within the allowable geometric limits, thus excluding non-conformity as a source of modal discrepancy. The detailed distribution of deviations is presented in the following section. Dimensional deviation at each measurement point was quantified using:

(1) $${\mathrm{Deviation}}_{\mathrm{point}}=\left(\frac{\mathrm{Measured}\;\mathrm{Value}}{\mathrm{Nominal}\;\mathrm{Value}}-1\right)\times 100$$

**Figure 2:**
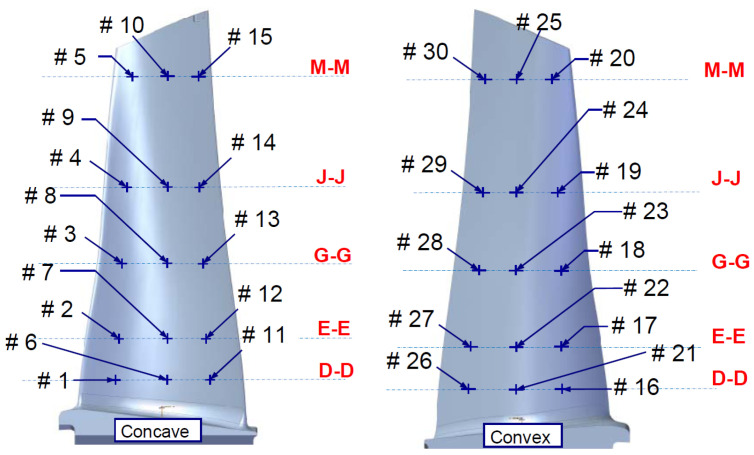
Location of measuring points: From #1 to #15 on the concave side of the blade, from #16 to #30 on the convex side of the blade. Groups of six points, three on each side, have been measured at the same longitudinal location on the blade, indicated by the double letters.

**Figure 3:**
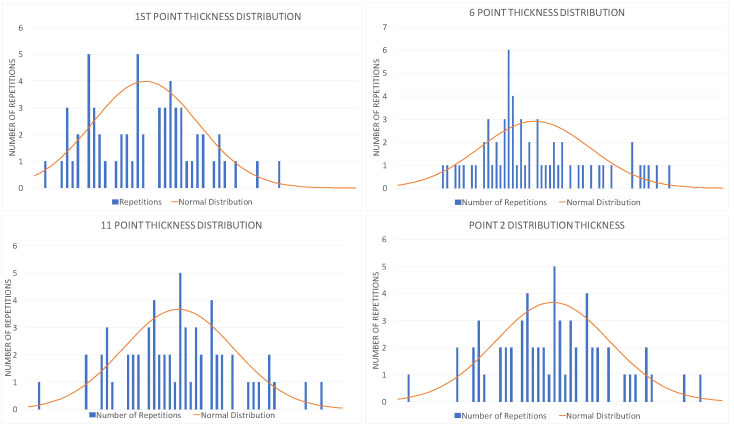
Graphs of deviations of four points, given as example. The thickness, measured over the thirty points considered in the study, is considered as wall thickness and compared with nominal design values.

For each reference point on the pressure and suction sides (the index of points can be observed in Figure 2), a histogram of the deviation distribution was generated, accompanied by the corresponding ideal normal distribution for comparison. The abscissa represents the magnitude of the deviation (negative or positive, from smaller to bigger), while the ordinate represents the number of blades exhibiting that specific deviation (the number of repetitions). A series of four graphs out of the thirty analyzed and produced are provided in Figure 3. Figure 3 presents the statistical distribution of thickness deviations for the 61 inspected blades, when compared to the nominal value, that comes from the original design (CAD). The deviation was calculated and computed according to Equation (2). The experimental data display an approximately normal distribution, with the fitted Gaussian curve (visualized in orange) providing a satisfactory qualitative representation of the measured scatter. This behavior suggests that the dimensional variability is mainly random in nature and not indicative of systematic manufacturing deviations. Although a formal goodness-of-fit metric was not evaluated at this stage, the visual agreement between the histogram and the fitted normal distribution supports the assumption of random variability. This visualization provided rapid screening for outliers and manufacturing trends. Across all evaluated points, the distributions were centered around the nominal value and showed no occurrences outside the acceptance range. This confirms that manufacturing variability was well-controlled and that no blade in the dataset should be excluded from dynamic testing on dimensional grounds. Importantly, the absence of geometric non-conformities suggested that geometry alone could not account for the discrepancies between calculated and measured modal frequencies. Consequently, subsequent analyses focused on other sources of variation, including constraint modeling, material anisotropy, and the role of internal features such as turbolators.

### 2.3. Influence of Geometrical Deviations on Modal Frequencies

To assess whether manufacturing deviations could explain the dispersion observed in the experimentally measured natural frequencies, a correlation study was conducted between local geometric deviations and the corresponding modal frequency deviations of each tested blade. The deviation was intended as the difference in thickness of the profile from the value considered in the CAD (the original model of the blade). The analysis was performed for both René 80 and DS nickel-based blades, using the measured frequencies of the first four vibrational modes. Geometrical deviation at point *i* was computed as:

(2) $${\mathrm{Deviation}}_{\mathrm{point}}=\left(\frac{\mathrm{Measured}\;{\mathrm{Value}}_{\mathrm{point}}}{\mathrm{Nominal}\;{\mathrm{Value}}_{\mathrm{point}}}-1\right)\times 100$$

Frequency deviation for blade b and mode m was defined as:

(3) $${\mathrm{Deviation}}_{f,m}=\left(\frac{f_{m}^{\left(\mathrm{meas}\right)}}{f_{m}^{\left(\mathrm{avg}\right)}}-1\right)\times 100,\;m=1,2,3,4$$

where f_m_^avg^ is the batch-average frequency for mode m. To investigate local and regional effects, geometrical deviations were also aggregated over groups of points expected to influence modal stiffness:

(4) $${\mathrm{Deviation}}_{G}=\sum_{i\in G}{\mathrm{Deviation}}_{i}$$

Grouped sets included spanwise bands, leading-edge and trailing-edge subsets, and full-section aggregates.

#### 2.3.1. Single-Point Deviation Analysis (René 80 Blades)

Single-point deviation values were plotted against mode-specific frequency deviations. Linear regression was applied to evaluate potential trends. Across all inspected points—especially those located near the root, which are most influential for the predominantly flexural first mode—no statistically meaningful correlation was observed. Regression slopes were consistently near zero, and data scatter dominated any apparent trend. This result indicates that local dimensional variation, at the magnitude present in the manufactured blades, does not significantly affect modal frequencies.

#### 2.3.2. Grouped Deviation Analysis (René 80 Blades)

Based on mode-shape characteristics, deviation groups were constructed to represent regions of similar structural influence (e.g., lower-span band, mid-section band, full-section sum, leading edge, trailing edge). Each grouped deviation metric was compared to the frequency deviation of modes 1–4. Again, no systematic correlation emerged. Although minor slopes were visible in some regression fits, the data dispersion was significantly larger than the trend lines, indicating that any observed inclination was not functionally meaningful. Consequently, geometric deviation, either local or regional, is not a determining factor in the modal frequency variability of the René 80 blades.

#### 2.3.3. Deviation Analysis for DS Nickel-Based Blades

**Figure 4:**
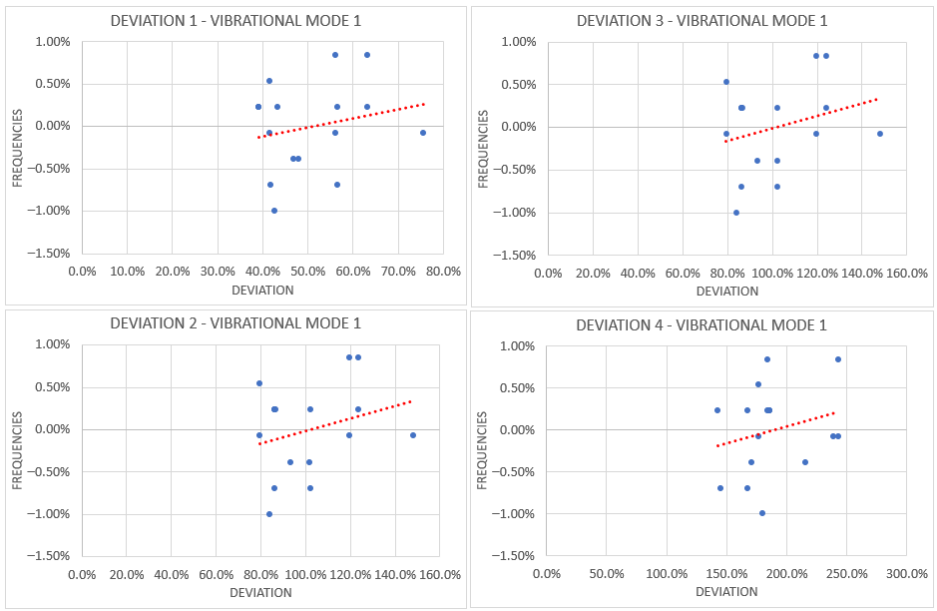
Four Graphs were produced out of all, in order to obtain results regarding the value of deviation for vibrational mode 1 (as an example, but were studied for every single mode up until the eighth). As it can be clearly seen, the scatter associated with the measures of the deviation from nominal value is far smaller than the deviation currently investigated, thus not having a big impact on final values.

The same procedure was applied to the batch of DS blades, and as reference, four graphs out of the batch produced is listed in Figure 4. Figure 4 illustrates the relationship between grouped geometric deviations and the corresponding frequency deviations for the first vibrational mode (Mode 1). A weak dependence is observable in all considered configurations, as indicated by the fitted linear regressions. However, the scatter of the data points still remains relatively large when compared to the magnitude of the slope, indicating that the observed variability in frequency is only slightly influenced by the considered geometric groupings (and therefore by geometrical factors). Although a limited dependence between deviation magnitude and frequency shift is present, the effect remains small relative to the overall dispersion of the data. As a result, this variability was not deemed sufficiently influential to justify further refinement or corrective modeling, and the frequency scatter is instead attributed primarily to combined modeling uncertainties and material-related effects.

This reinforced the conclusion that manufacturing tolerances were sufficiently tight, and that discrepancies between measured and numerically predicted frequencies must arise from other sources, not geometry. After this preliminary analysis it was find that all blades were geometrically compliant with design tolerances. Neither single-point deviations nor grouped deviations exhibited meaningful correlation with modal frequency shifts. Modal scatter must therefore be attributed to non-geometric factors, including:

- constraint modeling and broach-block compliance,
- material property uncertainty (especially anisotropy in DS blades),
- internal features such as turbolators.

This justified shifting the focus of the investigation towards material modeling and structural details rather than manufacturing geometry.

## 3. Effect of the Rib Turbolators

Rib turbulators are small internal protrusions commonly employed in gas turbine blades to enhance convective heat transfer between the internal cooling air and the blade material, typically a nickel-based superalloy. By deliberately promoting turbulence within the internal cooling passages, rib turbulators induce boundary-layer separation and reattachment, thereby increasing turbulence intensity and the associated heat transfer coefficient. This mechanism is essential for maintaining acceptable metal temperatures in high-pressure and high-temperature turbine stages, where thermal loading represents a primary life-limiting factor. To provide the reader with a comprehensive understanding of the experimental procedure, a brief overview of the testing methodology is presented. To determine the blade frequencies, each blade was secured in a broach block using a pneumatic clamping system. Once properly fixed, the blade was impacted with a hammer to induce vibrational response. The resulting vibration frequencies were recorded using a microphone and subsequently plotted for analysis and visualization.

From a structural perspective, rib turbulators are non-load-bearing features whose characteristic dimensions are significantly smaller than those of the blade. However, their presence modifies the internal mass distribution and local stiffness, which may influence the dynamic response of the component. Consequently, their effect cannot be neglected when high-fidelity modal predictions are required.

In the present study, rib turbulators were initially omitted from the finite element model to reduce geometric complexity and improve numerical efficiency during the preliminary analysis phase. This simplification facilitated a clearer evaluation of the influence of material properties, boundary conditions, and geometric deviations on the natural frequencies. Subsequently, rib turbulators were reintroduced into the model to quantify their impact on the vibrational behavior of the blade and to assess the validity of the simplified modeling assumptions. In order to correctly compute the value of the deviation from the measured values, the following Formula (5) was used, and repeated for every single mode up to the eighth;

(5) $${\mathrm{Deviation}}_{frequency}=\left(\frac{f^{\left(calculated\right)}}{f_{avg}^{\left(measured\right)}}-1\right)\times 100$$

**Figure 5:**
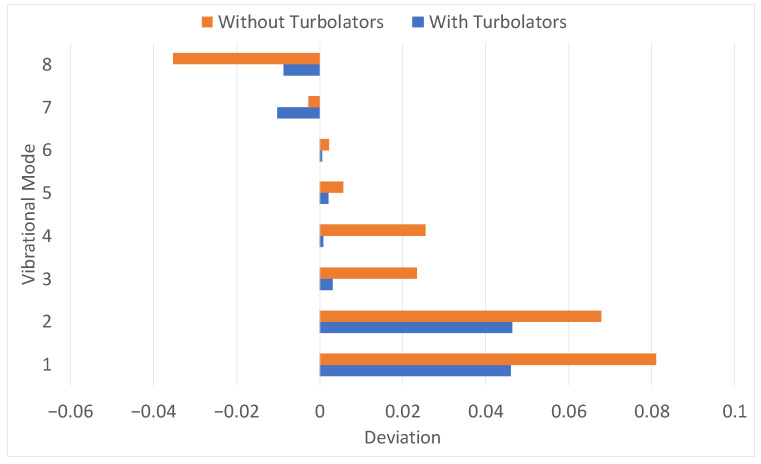
Comparison between the previous results (without turbolators) and with turbolators. As it can be seen the deviation is reduced for all vibrational modes.

The effect of the introduction of the Rib Turbolator’s effect can be easily visualized in Table 1, and a comparison between the situation before and after including those parts can be observed in Figure 5.

These results highlight how the explicit inclusion of rib turbolators in the finite-element model plays a fundamental role in reducing the previously more significant discrepancy between measured and calculated frequencies. The presence of such internal appendices, often neglected in simplified blade representations, such as in the beginning section of the work, introduces minimal local stiffness but non-negligible mass contributions that significantly affect the dynamic response of the component. By accounting for these features, present in the original CAD model and in the real-world component, the numerical model achieves a closer-to-reality evaluation of the modal frequencies, particularly for the lower-order modes, which are known to be more sensitive to global stiffness distribution and are the easiest for the operator to measure. The addition of rib turbolators enables for a finer and more accurate assessment of the frequency deviations observed during the experimental campaign, improving the overall fidelity of the simulation and reinforcing the importance of detailed geometric representation during the validation phase of the blade development process. This improvement is reflected in the systematic reduction of frequency errors, confirming that discrepancies previously attributed to material behavior or boundary condition modeling can, in part, be explained by the omission of internal geometric features. Consequently, the inclusion of turbolators represents a fundamental step toward achieving a physically consistent and reliable dynamic characterization, ultimately rendering the calculated results more representative of the real blade behavior. For the aforementioned reasons, it was deemed useful to include turbolators for all the following considerations and results.

## 4. Grain Orientation Effects

Directionally solidified nickel-based superalloys exhibit pronounced anisotropic mechanical and modal behaviors due to their preferential growth direction of columnar grains along the solidification pattern. As a consequence, the elastic response of turbine blades manufactured using this process is inherently very dependent on the orientation of the crystallographic axes with respect to the global blade reference system. This aspect becomes particularly relevant in dynamic analyses, where even moderate variations in stiffness distribution can lead to measurable changes in natural frequencies and mode shapes. In the present study, grain orientation was systematically varied in order to assess its influence on the vibrational behavior of the blade. The investigation was conducted by rotating the material principal axes with respect to the blade coordinate system, while maintaining identical geometry, boundary conditions, and loading assumptions. This approach allowed the isolation of anisotropic material effects from other contributing factors, such as geometric deviations or constraint modeling.

**Figure 6:**
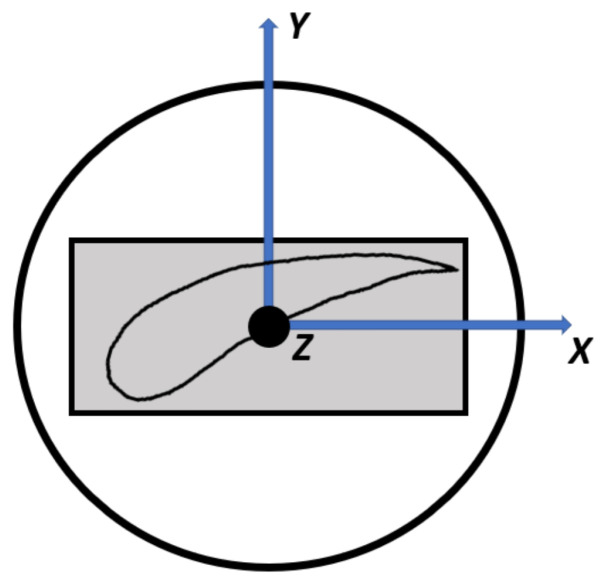
The axis orientation when considering the blade (here represented in grey) with each axis viewed from above. This is the same orientation of coordinate system used for the assessment and calculations.

**Figure 7:**
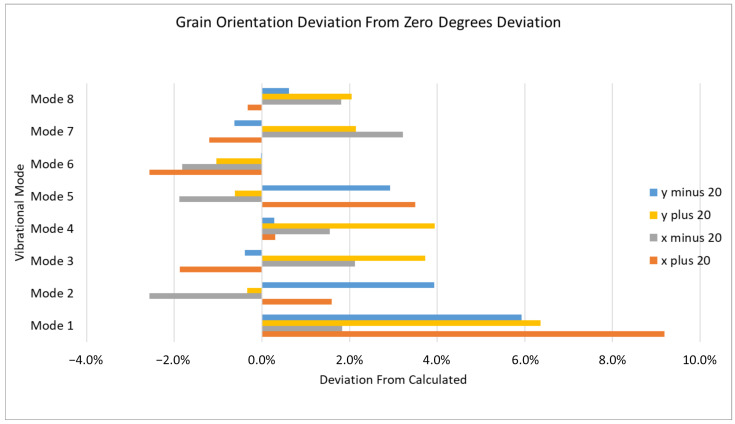
The deviations for every value of angle variation for every axis. It can be clearly observed how all the values of orientation do not optimize a single mode in particular.

**Figure 8:**
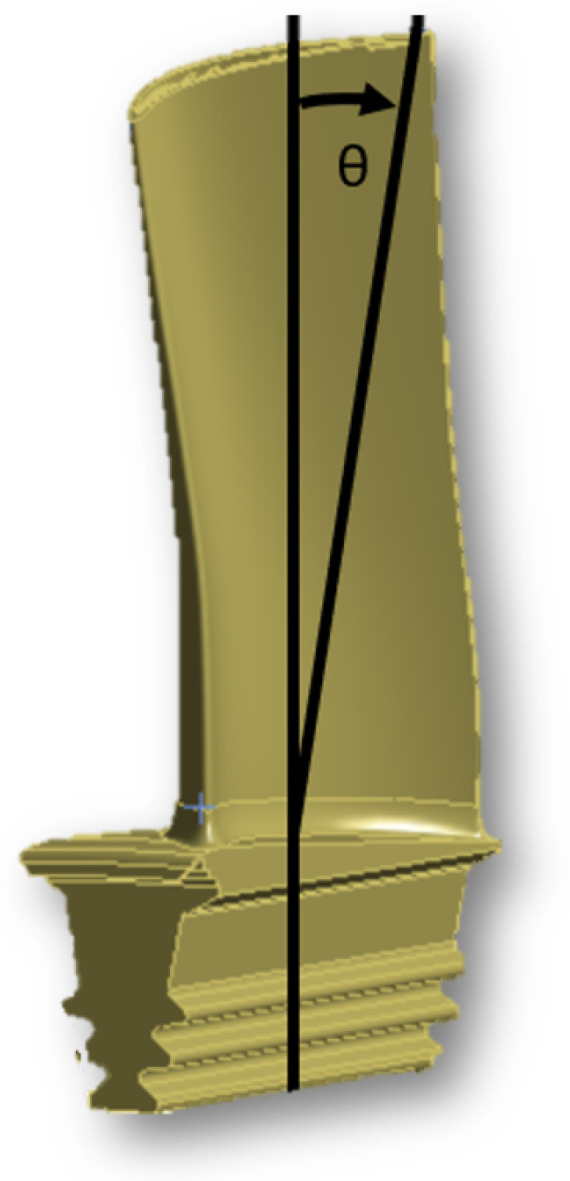
A representation of the blade with a variation on the value of the angle indicated θ.

Grain orientation is defined using the longitudinal axis of the blade as the *z*-axis, the axial axis as the *x*-axis, and a right-handed coordinate system to define the *y*-axis, as visible in Figure 6. Orientation uncertainty is subsequently modeled by rotating the material coordinate system (since the material is not isotropic) within a plus or minus 20° angle, defined as θ, following the right-hand convention; the modified orientation is obtained by applying a rotation of ±20° around the reference axis, following, as mentioned before, the right-hand convention, which implies a subsequent rotation of the plane defined by the remaining two axes around that reference axis. The results (observable in Figure 7) demonstrate that grain orientation has a non-negligible impact on the calculated natural frequencies, with deviations increasing as the orientation departs from the reference alignment. In particular, rotations about the principal axes produced asymmetric frequency shifts, reflecting the directional dependence of the elastic moduli. Lower-order modes were generally less sensitive to orientation changes, whereas higher-order modes exhibited increased variability due to their stronger dependence on local stiffness distributions. These findings highlight the importance of accurately defining grain orientation when modeling directionally solidified turbine blades. While simplified isotropic assumptions may be acceptable for preliminary analyses, high-fidelity correlation with experimental data requires explicit consideration of anisotropic material behavior. The inclusion of grain orientation effects therefore represents a necessary step toward improved predictive capability and reliable dynamic characterization. In order to asses the values of maximum influence, as rotational values for axes, the maximum acceptable extremes were considered. In particular it can be said how a variation of 20° is the maximum acceptable limit, hence the results show how the maximum variation affects vibrational frequencies and vibrational modes. The variation is calculated by rotating around a single axis as shown in Figure 8. In order to more clearly grasp how the orientation is defined, it was needed to observe the blade from above and clearly underline each axis, such as in Figure 6.

## 5. Simplified Optimization

Despite the use of high-fidelity geometric models derived directly from the original CAD data, realistic boundary conditions (both infinitely rigid and broach-block constrained), and fully anisotropic material definitions, a residual discrepancy between experimentally measured and numerically predicted natural frequencies persisted. This non-negligible mismatch indicated that uncertainties in the effective elastic properties of the material could represent a significant source of error. In particular, nominal values of Young’s modulus *E* and shear modulus *G*, typically obtained from material databases or supplier specifications, may not fully reflect the actual behavior of cast and heat-treated turbine blades.

To address this issue, an optimization strategy was implemented with the objective of tuning the elastic properties to improve agreement with experimental modal data, focusing on the first two vibrational modes, which are the most critical for validation purposes. The approach was formulated as an inverse problem in which the elastic moduli were iteratively adjusted to minimize the deviation between computed and measured natural frequencies. The optimization was performed using a dedicated in-house numerical routine, while the finite element model and boundary conditions were kept unchanged to ensure consistency with previously established results. The optimization process demonstrated that relatively modest variations in the elastic constants can produce significant improvements in frequency prediction, particularly for the lowest modes. A progressive reduction in frequency deviation was achieved across successive iterations, indicating both convergence and robustness of the procedure. The resulting optimized elastic moduli remained within physically plausible ranges, confirming that the adjustments represent an effective material characterization rather than a numerical artifact. These findings highlight material property tuning as a powerful tool for enhancing the predictive capability of finite element models during the validation phase. Although the optimized properties should not be interpreted as universal material constants, they provide an effective representation of blade behavior under the specific manufacturing and testing conditions considered. This methodology therefore offers a practical framework for reconciling numerical simulations with experimental observations in industrial turbomachinery applications.

Two distinct optimization routes were investigated. Given the anisotropic nature of the material, characterized by independent Young’s (**E**) and shear moduli (**G**) in different directions, the initial approach consisted of independently optimizing the elastic constants by formulating an algebraic system based on the first four natural frequencies. Firstly, two dimensionless auxiliary parameters related to the elastic and shear modulus were defined as follows,

(6) $$\left\{\begin{array}{cc} \zeta & =\frac{\sqrt{E}-\sqrt{E_{1}}}{\sqrt{E_{2}}-\sqrt{E_{1}}}\\ \eta & =\frac{\sqrt{G}-\sqrt{G_{1}}}{\sqrt{G_{2}}-\sqrt{G_{1}}} \end{array}\right.$$

where the subscripts 1 and 2 refer to two tentative values of the modulus (both for Young’s Module and Shear Module). Then, the natural frequencies were assumed to depend linearly on only one of the auxiliary parameters. This assumption was developed by observing that the first mode is mainly driven by the elastic modulus along the *x*-axis, the second by the elastic modulus along the *z*-axis, and the third and fourth by the shear modulus in the *yz*- and *xy*-planes, respectively. Consequently, the following set of four equations can be derived

(7) $$\left\{\begin{array}{cc} f_{m}^{I} & =f_{1}^{I}\left(1-\zeta_{x}\right)+f_{2}^{I}\left(\zeta_{x}\right)\\ f_{m}^{II} & =f_{1}^{II}\left(1-\zeta_{z}\right)+f_{2}^{II}\left(\zeta_{z}\right)\\ f_{m}^{III} & =f_{1}^{III}\left(1-\eta_{x}\right)+f_{2}^{III}\left(\eta_{x}\right)\\ f_{m}^{IV} & =f_{1}^{IV}\left(1-\eta_{z}\right)+f_{2}^{IV}\left(\eta_{z}\right) \end{array}\right.$$

where the frequencies *f*_1_^i^ and *f*_2_^i^ refer to the natural frequencies of mode *i*-th computed with the finite element model and the tentative modulus 1 and 2, respectively, while *f*_m_^i^ is the measured natural frequencies of mode *i*-th. Solving Equation (7) for the auxiliary parameters yields the following

(8) $$\left\{\begin{array}{cc} \zeta_{x} & =\frac{f_{m}^{I}-f_{1}^{I}}{f_{2}^{I}-f_{1}^{I}}\\ \zeta_{z} & =\frac{f_{m}^{II}-f_{1}^{II}}{f_{2}^{II}-f_{1}^{II}}\\ \eta_{x} & =\frac{f_{m}^{III}-f_{1}^{III}}{f_{2}^{III}-f_{1}^{III}}\\ \eta_{z} & =\frac{f_{m}^{IV}-f_{1}^{IV}}{f_{2}^{IV}-f_{1}^{IV}} \end{array}\right.$$

The target elastic and shear modulus were derived once the auxiliary parameters had been computed,

(9) $$\left\{\begin{array}{cc} E_{x}^{t} & ={\left(\zeta_{x}\left(\sqrt{E_{2x}}-\sqrt{E_{1x}}\right)+\sqrt{E_{1x}}\right)}^{2}\\ E_{z}^{t} & ={\left(\zeta_{z}\left(\sqrt{E_{2z}}-\sqrt{E_{1z}}\right)+\sqrt{E_{1z}}\right)}^{2}\\ G_{x}^{t} & ={\left(\eta_{x}\left(\sqrt{G_{2x}}-\sqrt{G_{1x}}\right)+\sqrt{G_{1x}}\right)}^{2}\\ G_{z}^{t} & ={\left(\eta_{z}\left(\sqrt{G_{2z}}-\sqrt{G_{1z}}\right)+\sqrt{G_{1z}}\right)}^{2} \end{array}\right.$$

This procedure is iterative because, contrary to the initial assumption, the computed natural frequencies *f*_1_^i^ and *f*_2_^i^ depend on all the modulus *E*_x,z_ and *G*_x,z_.

**Figure 9:**
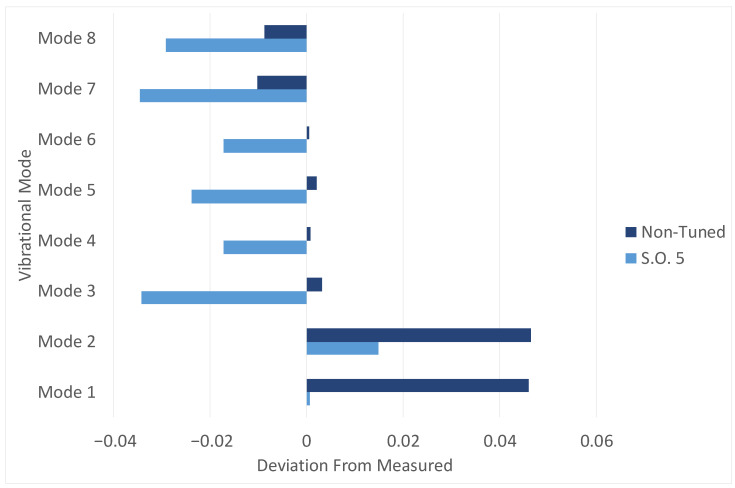
The graph showing a comparison between the non-tuned case and the last iteration of the Algebraic System Method. The “SO” stands for Simplified Optimization.

The solutions that emerged were acceptable, as shown in Figure 9 and for more detail in Table 2, but not accurate or resolutive enough. In addition, an important aspect emerged: when considering the first two frequencies, the shear modulus values did not significantly influence the frequencies of the first two modes. Therefore, it was decided to concentrate on the two independent Young’s modulus values for a more refined optimization code.

## 6. Full Optimization

A second, more advanced optimization strategy was implemented through the development of a dedicated Young’s modulus calibration routine based on an error minimization framework. The objective function was formulated using experimentally measured natural frequencies, together with numerical predictions from previous simulations and the initial optimization stage, thereby establishing a high-fidelity reference dataset for the calibration process. The resulting nonlinear least-squares problem was solved using the Levenberg–Marquardt algorithm, which ensured efficient and robust convergence toward the optimal set of elastic parameters.

**Figure 10:**
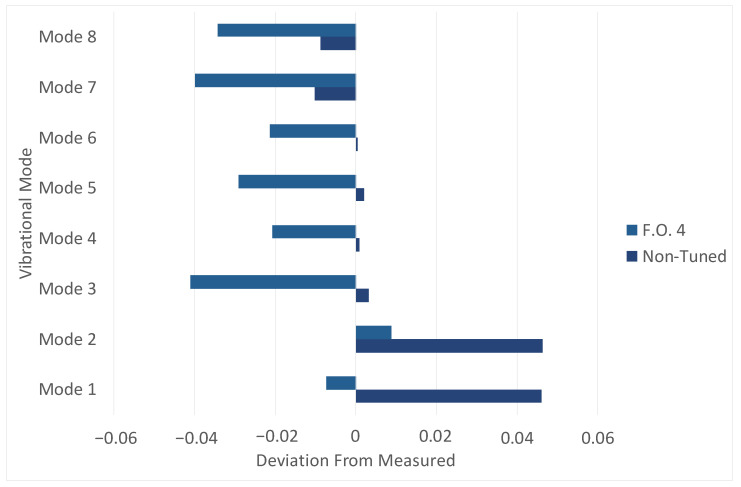
The final deviation, after the fourth iteration of the optimization code, compared with the René 80 situation and the non-tuned case.

This approach was motivated by the limitations of the preceding method, in which the coupled influence of independent Young’s moduli was partially neglected for simplification. In contrast, the present formulation explicitly accounted for the simultaneous contribution of both elastic moduli to the first two natural frequencies, ensuring that cross-effects between material parameters and modal response were fully captured. The application of this optimization framework led to a substantial reduction in the residual discrepancies between the natural frequencies computed and measured. The optimized results closely matched the experimental targets and remained within the acceptability thresholds defined by Ansaldo Energia’s validation standards. Moreover, the use of a non-linear optimization solver proved essential for accurately capturing the coupled dependence of the modal response on the elastic properties, enabling the inverse problem to converge toward a physically consistent and more accurate solution. The final result is observable in Figure 10 and Table 3. The reference of the deviation is, as usual, the measured values of frequencies.

## 7. Discussion

The dynamic characterization of the turbine blade presented in this work demonstrates a progressive improvement in the agreement between numerical predictions and experimental measurements as successive enhancements in model fidelity were introduced. Initial finite element simulations, although based on high-quality CAD geometry and refined meshes, exhibited systematic deviations from experimentally measured natural frequencies, particularly for the lower-order modes. These modes are of primary importance, as they are associated with the highest vibrational energy and therefore dominate the dynamic response of the component.

The explicit inclusion of internal rib turbolators in the numerical model resulted in a measurable and consistent reduction in frequency discrepancies. Although rib turbolators are non-load-bearing features, their contribution to local mass distribution was shown to have a non-negligible influence on the modal response. Accounting for these internal structures significantly improved the predictive capability of the model, especially for the first two modes, confirming that simplified geometric representations are inadequate for accurate dynamic validation during the blade development phase.

Subsequently, the effect of material anisotropy was investigated by introducing fully anisotropic elastic properties for the directionally solidified alloy. A parametric study on grain orientation, within admissible manufacturing tolerances, was performed to assess its impact on predicted natural frequencies. While grain orientation was found to significantly affect modal behavior, no orientation configuration provided a systematic improvement in agreement with experimental data. This indicates that, although anisotropy is physically relevant and necessary for completeness, it is not sufficient on its own to reconcile numerical and experimental results, and the nominal 0° orientation remains the most representative configuration.

Despite the combined inclusion of high-fidelity geometry, realistic boundary conditions, internal features, and anisotropic material behavior, a residual mismatch between measured and computed frequencies persisted. This outcome highlighted uncertainties associated with the effective elastic properties of the material, which may differ from nominal database values due to casting, heat treatment, manufacturing variability, and the complex geometry of the blade. To address this limitation, an optimization-based model updating strategy was implemented in two successive phases. By formulating the problem as an inverse identification task, the elastic moduli were iteratively adjusted through an error-minimization procedure targeting the first two vibrational modes. The application of a non-linear least-squares optimization algorithm enabled a substantial reduction in frequency deviations, bringing numerical predictions within the acceptance thresholds required for industrial validation.

Overall, the results demonstrate that a hierarchical refinement approach, progressively incorporating internal geometry, anisotropic behavior, and optimized material properties, yields a numerical model that closely reproduces the actual dynamic behavior of the turbine blade. The proposed methodology provides a robust and physically consistent framework for modal validation across different materials and manufacturing conditions. The results are summarized in Table 4.

### Role of Experimental Validation

The experimental campaign conducted in this study proved essential for assessing the reliability and physical consistency of the numerical models developed for turbine blade dynamic characterization. The comparison between measured and calculated natural frequencies provided a clear benchmark for evaluating successive modeling assumptions and identifying dominant sources of discrepancy. In this context, experimental data served not only as a validation reference but also as a diagnostic tool guiding targeted model refinements.

The results confirmed that high-quality geometric representation and refined finite element discretization, while necessary, are not sufficient on their own to ensure accurate modal predictions. Experimental evidence emphasized the importance of accounting for internal features such as rib turbolators, whose influence on dynamic response would have been underestimated without direct measurements. Similarly, the experimental findings highlighted the limited yet non-negligible role of material anisotropy, justifying its inclusion for model completeness even when its corrective effect proved non-systematic.

Most importantly, experimental modal data enabled the successful implementation of an optimization-based updating strategy, allowing effective elastic properties to be identified within physically meaningful bounds. The resulting reduction in frequency deviations confirms that experimental modal analysis remains a fundamental component of blade validation workflows, particularly in the presence of manufacturing-induced variability. Overall, the experimental results validate the proposed methodology as a robust and industrially applicable framework for turbine blade dynamic assessment.

## 8. Conclusions

This study demonstrates that accurate dynamic characterization of gas turbine blades requires a hierarchical modeling approach that extends beyond refined geometry and mesh discretization. Experimental evidence shows that internal features, material anisotropy, and uncertainties in effective elastic properties play a decisive role in shaping the modal response, particularly for the lower-order modes that dominate vibrational behavior. While high-fidelity finite element models capture the overall trends, residual discrepancies persist unless these factors are explicitly addressed. By combining experimental modal analysis with progressive model refinement and optimization-based material tuning, numerical predictions were brought within industrial acceptance thresholds. The proposed methodology provides a robust and physically consistent framework for turbine blade validation and highlights the essential role of experimental data in achieving reliable dynamic predictions for advanced turbomachinery components.

## Figures and Tables

**Table 1:** Table of the deviations of frequencies for every Vibrational Mode (indicated in the table as “M”) with the turbolators when compared without them.

	M1	M2	M3	M4	M5	M6	M7	M8
Deviation	−3.2%	−2.1%	−1.6%	−3.5%	−0.2%	−0.2%	0.1%	0.8%

**Table 2:** The table with the results of the optimization of the values of E and G with the Algebraic System on the last iteration. “S.O.” in the table stands for Simplified Optimization, while the Vibrational Modes are indicated with the letter “M”.

Deviation	M1	M2	M3	M4	M5	M6	M7	M8
S.O.	0.1%	1.5%	−3.4%	−1.7%	−2.4%	−1.7%	−3.5%	−2.9%

**Table 3:** The summary table for the optimization of $E_{x}$ and $E_{z}$ with the code at the final iteration. It has to be noted how the values of deviation fall significantly into the range of acceptability of not only accurate literature, but also fit with René 80 values. The vibrational mode is indicated with “M”, while “F.O.” stands for “Full Optimization”. The “4” refers to the fourth iteration related to the code.

Deviation	M1	M2	M3	M4	M5	M6	M7	M8
F.O. 4	−0.73%	0.88%	−4.10%	−2.07%	−2.91%	−2.13%	−3.98%	−3.43%

**Table 4:** The summarised table of the results. The Rib Turbolators (R.T.) scenario refers to the values of deviation of frequency when considering the model before and after the inclusion of turbolators. The Simplified Optimization (S.O.) and Final Optimization (F.O) refer to the material optimization strategy.

Case	M1	M2	M3	M4	M5	M6	M7	M8
R.T.	−3.2%	−2.1%	−1.6%	−3.5%	−0.2%	−0.2%	0.1%	0.8%
S.O.	0.1%	1.5%	−3.4%	−1.7%	−2.4%	−1.7%	−3.5%	−2.9%
F.O.	−0.73%	0.88%	−4.10%	−2.07%	−2.91%	−2.13%	−3.98%	−3.43%

## Data Availability

The data presented in this study are available upon reasonable request from the corresponding author. The data are not publicly available due to confidentiality constraints associated with proprietary industrial data.
